# Early and late outcomes of component separation with transversus abdominis release with mesh augmentation versus primary suturing for the management of abdominal dehiscence: a retrospective comparative study

**DOI:** 10.1186/s13017-026-00690-2

**Published:** 2026-03-31

**Authors:** Tamer. A. A. M. Habeeb, Abdulzahra Hussain, Jose Bueno-Lledó, Mariano Eduardo Giménez, Alberto Aiolfi, Ahmed Abdelsamad, Massimo Chiaretti, Igor A. Kryvoruchko, Mallikarjuna N. Manangi, Mohamed Tag El-Din, Nasreldin Mohammed Algalaly, Mohamed Ibrahim Shalamesh, Mohamed Fathy Labib, Walid Rafat Abdelaty Abdelfattah, Mohammed Abbas, Mostafa Mahmoud Salama Mostafa, Mahmoud E. Nagaty, Mohamed Hassan Mohamed Elkaseer, Mahmoud Abd Alhady Abd Alaziz Abd Alhady, Ahmed Fayez Othman, Hamdi Elbelkasi, Mohamed Ibrahim Abo Alsaad, Mahmoud Moustafa AL-Shareef, Amr Khalil, Abouelatta K H. Ali, Maged Z. Youssef, Mohamed Adel Saqr, Mohamad Naeem Elnahas, Asmaa Mohamed Abdelhady, Mahmoud Abubakr Negm, Mohammed Barakat, Bassam Mousa, Alaa A. Fiad, Mahmoud Abdou Yassin, Mostafa M. Khairy, Tamer Wasefy, Ahmed M. El Teliti, Ahmed Salah Arafa, Hasnaa Metwally, Taha A. Biomy, Baher Atef, Adel Morsi, Mohamed Mahmoud Almeniawy, Mohamed Abdallah Zaitoun, Ahmed Attia Saleh, Mohamed Lotfy, Mohamed Mahmoud Mokhtar Mohamed, Abd Elwahab M. Hamed

**Affiliations:** 1 Department of General Surgery, Faculty of Medicine, Zagazig University, Zagazig, Egypt; 2 Sheffield University, Sheffield, UK; 3 University of Alkafeel, Najaf, Iraq; 4 Hospital Universitari i Politècnic la Fe, Valencia, Spain; 5 University of Strasbourg, Strasbourg, France; 6 IHU-IRCAD, Strasbourg, France; 7 Division of General Surgery, Department of Biomedical Science for Health, I.R.C.C.S. Ospedale Galeazzi-Sant’Ambrogio, University of Milan, Milan, Italy; 8 Department of Surgery II, University of Witten/Herdecke, Witten, Germany; 9 Department of General Surgery Specialties and Organ Transplant, Faculty of Pharmacy and Medicine, Sapienza Rome University, Rome, Italy; 10 Department of Surgery No. 2, Kharkiv National Medical University, Kharkiv, Ukraine; 11 Department of General Surgery, Bengaluru, India; 12 General Surgery Department, Faculty of Medicine, Al-Azhar University, Cairo, Egypt; 13 Department of General Surgery, Faculty of Medicine for Girls, Cairo Al Azhar University, Cairo, Egypt; 14 Mataryia Teaching Hospital (GOTHI), Cairo, Egypt; 15 General Surgery Department-Faculty of Medicine, Merit University, Sohag, Egypt; 16 Department of General Surgery, Tanta University, Tanta, Egypt; 17 General Surgery Department, Al-Ahrara Teaching Hospital, Zagazig, Egypt; 18 Misr University for Science and Technology, Cairo, Egypt; 19 General Surgery department, Faculty of Medicine, October 6 University, 6th of October City, Egypt; 20 Plastic and Reconstructive Surgery Department, Faculty of Medicine, Zagazig University, Zagazig, Egypt; 21 Plastic and Reconstructive Surgery Department, Faculty of Medicine, Shebin Teaching Hospital, Menoufia, Egypt; 22 Department of Gynecology and Obstetrics, Faculty of Medicine, Zagazig University, Zagazig, Egypt

**Keywords:** Abdominal dehiscence, Component separation, Transversus abdominis release, Tension suturing, Incisional hernia, Surgical site infection

## Abstract

**Background:**

Abdominal dehiscence (AD) is a serious postoperative complication associated with a high risk of morbidity. Traditional primary suture repair (PS) is a simple but biomechanically deficient procedure. This study compared the early and late outcomes of posterior component separation (CS) using the transversus abdominis release (TAR) technique with mesh augmentation (MA) and PS for AD management.

**Materials and methods:**

This retrospective study included 252 patients who underwent surgical repair for complete AD Bjork Grade 1 A between January 2014 and September 2020. The patients were divided into two groups: CS + TAR+MA (Group A, *n* = 107) and primary suture (PS) repair (Group B, *n* = 145). The primary outcome was short-term morbidity (within 90 days), including surgical site occurrence (surgical site infection [SSI], hematoma, and seroma), pneumonia, ileus, and recurrent AD (RAD). The secondary outcomes were the incidence and risk factors of IH after AD repair. The patients were followed up for 5 years. Statistical analysis was performed using Kaplan-Meier survival analysis and multivariate logistic regression.

**Results:**

The baseline characteristics of the two groups were comparable. Group A was associated with a longer median operative time (92 (88–100) vs. 89 (84–91) min, *p* < 0.001) and mean hospital stay (11.2 ± 1.9 vs. 5.8 ± 1.5 days, *p* < 0.001), and higher rates of seroma (22.4% vs. 11.0%, *p* = 0.01) and hematoma (3.7% vs. 0%, *p* = 0.01). The SSI rates were comparable between the two groups (7.5% vs. 4.1%, *p* = 0.2). The incidence of IH was significantly lower in Group A than in Group B (5.6% vs. 13.1%, *p* = 0.04). Kaplan-Meier analysis confirmed the superior long-term IH-free survival in Group A (log-rank test, *p* = 0.009). Group A also had a lower RAD rate (1.9% vs. 7.6%, *p* = 0.04). Multivariate analysis revealed that PS repair (OR 40.0, 95% CI 2.1–78.0; *p* = 0.01), SSI (OR 13.4, 95% CI 2.3–77.6; *p* = 0.004), pneumonia (OR 12.3, 95% CI 1.9–77.5; *p* = 0.007), high BMI (OR 2.9, 95% CI 1.06–4.1; *p* = 0.03), ileus (OR 16.6, 95% CI 2.2-121.9; *p* = 0.006), RAD (OR 10.7, 95% CI 1.5–73.3; *p* = 0.01), infected mesh (OR 14.6, 95% CI 1.8–117; *p* = 0.01), and old age (OR 1.07, 95% CI 1.006–1.15; *p* = 0.03) significantly increased the risk of IH after AD repair. Elevated serum albumin levels were protective (OR 0.1, 95% CI 0.0–0.7; *p* = 0.02).

**Conclusion:**

Group A repair for AD was associated with a significantly reduced risk of IH and RAD compared to PS. Despite a higher rate of initial complications, such as seroma and hematoma, Group A provided more durable and definitive reconstruction. PS repair confers a 40-fold increased risk of IH and should be reconsidered in favor of tension-free Group A management of AD.

**Supplementary Information:**

The online version contains supplementary material available at 10.1186/s13017-026-00690-2.

## Introduction

Abdominal dehiscence (AD) is an unintended partial or complete acute abdominal fascial dehiscence following abdominal surgery, with or without visceral evisceration, and usually occurs between the 6th and 15th postoperative days [1–2]. The incidence of AD ranges from 3.8% in elective surgeries to 45% in emergency surgery [2–6]. AD is a risk factor for increased morbidity, enterocutaneous fistulae, long hospital stays, urgent reoperation, intra-abdominal sepsis, increased healthcare costs, and impaired quality of life [3, 7–10], with mortality rates ranging from 5% to 45%. In the long term, BA is a major risk factor for incisional hernia (IH) [8, 11–12].

In the early postoperative period, urgent repair of the AD is essential to prevent evisceration and abdominal cavity infection and to reduce morbidity [13–14]. The challenging closure of AD results in heterogeneous procedures with various techniques and modifications [14–16]. Only small fascial defects, adherent bowel, or suboptimal patient status may favor a nonsurgical approach [14]. For decades, primary suturing (PS) has been the cornerstone of treatment, often employing mass-closure techniques with various suture materials and approaches. However, PS has been associated with a high recurrence of AD (up to 35%) and IH (4–37.5%) [12, 17–22]. Despite this, various studies have attempted to introduce techniques to reduce the incidence of recurrent AD (RAD) and IH [9–10, 23]. This emphasizes the need for further action to decrease complications following PS for managing AD, including posterior component separation (CS) with transversus abdominis release (TAR) [24]. CS provides a tension-free closure of large AD. The high RAD and IH rates following treatment with AD could support the addition of mesh repair [12, 18, 20, 25–30]; however, mesh infection has been reported as a complication [10, 12, 19–20, 27, 31–33]. CS in emergency settings is scarce; however, our previous study showed promising results with a low incidence of IH (8.9%) [34]. Over the past few years, the European Hernia Society (EHS) has developed guidelines to optimize AD care. EHS recommends mesh reinforcement whenever fascial closure is feasible, with the type and location of the mesh being assessed by the operating surgeon [10, 35–38].

Risk factors for AD have been previously reported, with surgical site infection, coughing or chronic lung disease, hypoalbuminemia, chronic steroid use, obesity, linea alba thickness, and rectus diastasis being the most significant risk factors [8, 39–43].

To address this critical knowledge gap regarding the optimal treatment of AD, this study aimed to provide the first comparative analysis of short- and long-term outcomes following two surgical techniques: CS + TAR+MA and primary suture (PS).

## Methods

### Study design and patient eligibility

**Fig. 1:**
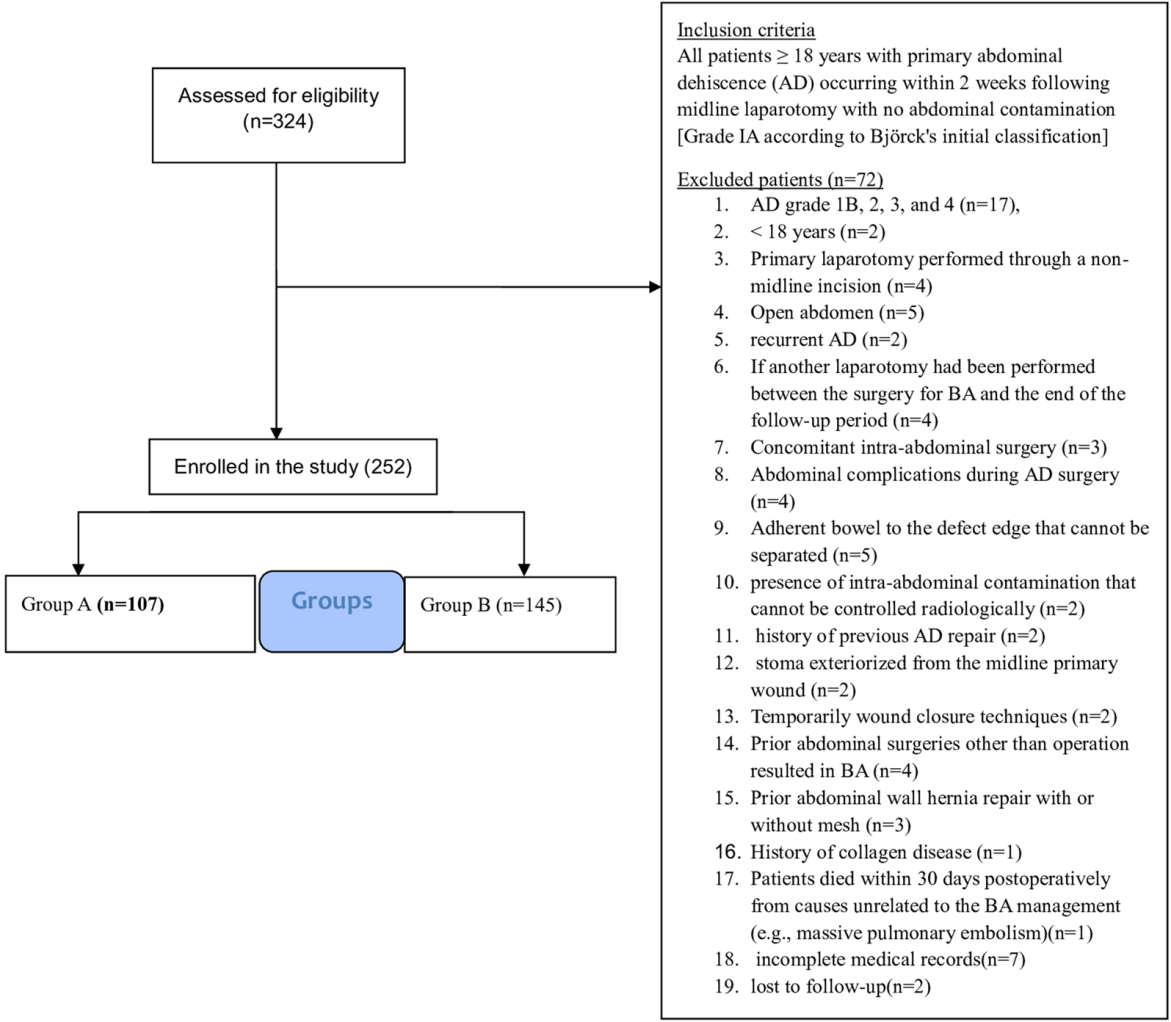
Flow chart of inclusion and exclusion criteria

This retrospective cohort study was conducted at our hospital between January 2014 and September 2020. This study was conducted in accordance with the Declaration of Helsinki and the Strengthening the Reporting of Observational Studies in Epidemiology (STROBE) guidelines and was registered at ClinicalTrials.gov (NCT07229703). The medical records of all adult patients (≥ 18 years) who underwent PCS + TAR+MA or primary suture with tension sutures for complete AD Bjork Grade 1 A [44] were enrolled. Panniculectomy was not performed in this study. Figure 1 shows the flowchart of the inclusion and exclusion criteria.

### Participating centres and surgeon experience

Subspecialized emergency surgical teams performed the procedures and adhered to a well-defined strategy for high-risk emergency surgical patients, including standardized preoperative [45], intraoperative [46], and postoperative [47] protocols, as well as documented standards for the surgical treatment of AD [10]. The success of the CS + TAR+MA technique depends on the learning curve. Our institutions support surgical training and ensure consistent availability of appropriate mesh materials. The surgeon’s necessary experience was calculated to be at least 16 mesh repairs [48], which all operating surgeons had. Patients with complete clinical data from five affiliated hospitals in Egypt [three academic centers: Zagazig, Al-Azhar, and Tanta universities; and two non-academic centers: Al-Ahrara Teaching Hospital and Shebin Teaching Hospital] were enrolled in this study.

### Definitions and outcomes measurement

The primary outcome was short-term morbidity (within 90 days), including surgical site occurrence (surgical site infection [SSI], hematoma, or seroma), pneumonia, ileus, and RAD. The secondary outcomes were the incidence and risk factors of IH after AD repair. Complete AD is characterized by fascial dehiscence, open skin, and evisceration [49]. Bjork Grade 1 A of AD: AD without visceroparietal adhesions or fixity of the abdominal wall, clean wound without contamination, and absence of enteral fistula [44]. This study included some of the patients described in our previous study [34]. Only patients who met the study’s enrollment criteria and had a complete 5-year follow-up from 5 recruitment centers were included. No patients were excluded based on mortality. The fascial closure procedure was performed as early closure in the first 4–7 days and late closure 7 days after the index operation [50]. According to the EHS, definitive fascial closure occurs when the fascia is completely closed with no remaining fascial defect (fascia-to-fascia closure) [10]. In our centers, diagnosed AD is always treated surgically, and if fascial closure is not possible during the first operation, negative-pressure wound therapy (NPWT) is used until fascial closure becomes feasible. The fascia defect was defined as the maximum distance between the bilateral rectus abdominis sheaths on preoperative abdominal computed tomography (CT). We defined short-term wound complications as any surgical site occurrence (SSI, seroma, wound dehiscence, or enterocutaneous fistula) within the first 90 days after surgery for AD [30, 51]. SSI was defined according to Centers for Disease Control and Prevention criteria and classified as superficial, deep, or organ-space infection [52–53]. Hematoma and seroma are diagnosed upon wound reopening, clinical assessment, or radiologic evaluation [30]. Recurrent AD (RAD) is the recurrence of AD after surgical treatment for this complication and is otherwise defined as above. This included tearing of the mesh after mesh repair, with resultant evisceration, and the need for subsequent (mesh) repair [30]. IH was defined as any abdominal wall gap with or without a bulge at the site of a postoperative scar or palpable by clinical examination and/or imaging [ultrasonography/CT scan] during follow-up [10, 54–55].

### Variables collected

Data were extracted from electronic medical records and included age, sex, BMI, ASA score, comorbidities (diabetes mellitus-DM, hypertension-HT, COPD), smoking, steroid use, family history of hernia, serum albumin level, time from index surgery to AD, departmental origin of the AD, possible etiology, dimensions of the fascial defect (cm), operative time (minutes), estimated blood loss (ml), need for transfusion, length of hospital and ICU stay (days), complications graded by the Clavien-Dindo classification [56], specific complications (surgical site infection-SSI, seroma, hematoma, pneumonia, ileus, myocardial infarction, pulmonary embolism), RAD, mortality, and IH.

### Surgical techniques and patient choice

Patients were allocated into two groups according to the surgical technique used for definitive fascial closure: Group A (CS + TAR+MA Group), as previously described [34, 57–59]. Two grams of cefuroxime were administered during anesthesia induction. Ethicon polypropylene mesh (30 × 30 cm, Prolene brand, square with PMH code) was inserted in the retro-muscular space and extended beyond the TAR. Group B (PS group): This procedure has been previously described in detail [10]. After exploration and debridement, the abdomen was closed using a mass closure technique with a continuous monofilament nonabsorbable suture (No. 1 polypropylene) involving all layers of the abdominal wall, except subcutaneous fat and skin, maintaining a suture-to-wound length ratio of at least 4:1 using “big bites” (3 cm) in “small steps” (5 mm). No mesh was used for this group. Self-locking knots were not used in this study. Retention sutures were then placed in this group. All surgeons in our departments strictly followed a common technique. The choice of technique was at the surgeon’s discretion and was influenced by patient stability, tissue quality, and the surgeon’s expertise.

### Postoperative follow-up

The patients were instructed to practice incentive spirometry and deep-breathing exercises postoperatively to reduce the risk of pneumonia and minimize coughing. If a cough was present, abdominal support was used during the episodes. Activities that suddenly increase intra-abdominal pressure, such as lifting heavy objects, straining during defecation or urination, vigorous coughing, or vomiting, for at least 2 months postoperatively. Stool softeners and good hydration are crucial to avoid constipation. An experienced surgical staff followed up the wound to detect signs of infection (redness, swelling, pain, and draining pus), seroma, hematoma, and wound dehiscence. According to our institutional protocol, antibiotic therapy was administered for emergency abdominal surgery. In the absence of signs of infection, antibiotics were continued for 4 days postoperatively [Cefuroxime 1.5 g IV q8h + Metronidazole 500 mg IV q8h]. If SSI was diagnosed, antibiotic duration was guided by clinical response, wound culture results, and the surgeon’s judgment, typically up to 8 days after SSI control [60]. Good nutrition, including adequate protein, is important for maintaining serum albumin > 3.5 g/dl. Smoking cessation is recommended because tobacco hinders wound healing and increases the incidence of respiratory complications. Rapid treatment for ileus or urinary retention using a nasogastric tube or a Foley catheter. Abdominal binders are recommended per institutional protocol. Outpatient follow-up visits were scheduled at 1, 3, 6, and 12 months and annually thereafter (follow-up period is 5 years), with clinical examination for IH and, if indicated, ultrasonography or CT.

### Statistical analysis

Statistical analysis was performed using SPSS version 27.0 (IBM Corp.). Continuous variables are expressed as mean ± standard deviation (SD) or median with interquartile range (IQR) based on normality, assessed using the Shapiro-Wilk test. Group comparisons were performed using the independent Student’s t-test or Mann-Whitney U test. Categorical variables are presented as numbers (percentages) and were compared using the Chi-square or Fisher’s exact test. IH-free survival was analyzed using the Kaplan-Meier method, and the groups were compared using the log-rank test. Univariate logistic regression was performed to identify variables associated with IH development. Variables with a p-value < 0.2 in the univariate analysis were entered into a multivariate logistic regression model to identify independent predictors. The selection of variables in the model was based on knowledge and clinical experience to improve the model. In our experience, we considered variables (age, sex, surgical technique, vertical length of AD, high serum albumin, BMI, operative time, SSI, seroma, pneumonia, ileus, RAD, and infected mesh) that are expected to predispose to incisional hernia. Each variable was evaluated while controlling for potential and well-known confounders using the ENTER method. Each variable was evaluated separately because we had a low incidence of incisional hernia (25 patients), which did not allow many predictors to be included in a single model and may have led to a non-robust estimate. In addition, multicollinearity is a well-known problem in the presence of multiple predictors. Multicollinearity can destroy a regression model and reverse the effects of predictors on the outcome. The results are reported as odds ratios (OR) and adjusted odds ratios (aOR) with 95% confidence intervals (CI). Statistical significance was set at a two-tailed *p*-value of < 0.05.

## Results

A total of 252 patients with AD stage 1 A who underwent surgical repair were enrolled in this study. Of these, 107 patients (42.5%) received CST + TAR+MA (Group A), and 145 patients (57.5%) underwent PS (Group B).

### Patient demographics and preoperative characteristics

The baseline and preoperative characteristics are shown in Table 1. In both groups, there were no statistically significant differences in age (*p* = 0.8), sex (*p* = 0.6), ASA score (*p* = 0.2), Time from index surgery till occurrence of AD (*p* = 0.6), BMI (*p* = 0.6), DM (*p* = 0.4), HTN (*p* = 0.1), COPD (*p* = 0.1), smoking (*p* = 0.3), steroid use (*p* = 0.2), serum albumin (*p* = 0.08), or anatomical site of the AD (*p* = 0.9).

**Table 1:** Patient demographics and preoperative characteristics in both groups

	Group A (CS + TAR+MA) (*n* = 107 )	Group B (primary suture) (*n* = 145)	*p*-value
Age (median, IQR)	41 (33–48)	42 (33.5–47)	0.8
Sex			
Male	44 (41.1%)	56 (38.6%)	0.6
Female	63 (58.9%)	89 (61.4%)	
ASA			
ASA-I	72 (67.3%)	112 (77.2%)	0.2
ASA-II	23 (21.5%)	23 (15.9%)	
ASA-III	12 (11.2%)	10 (6.9%)	
Time from index surgery till occurrence of AD (in days) (median, IQR)	7 (4–9)	7 (4–9)	0.6
BMI	33.9 ± 3.8	33.8 ± 3.6	0.6
DM	23 (21.5%)	26 (17.9%)	0.4
HTN	23 (21.5%)	20 (13.8%)	0.1
COPD	12 (11.2%)	9 (6.2%)	0.1
Smoker	25 (23.4%)	27 (18.6%)	0.3
Steroid	7 (6.5%)	5 (3.4%)	0.2
Family history of hernia	16 (15%)	22 (15.2%)	0.9
Serum albumin			
< 3.5gm%	28 (26.2%)	25 (17.2%)	0.08
≥ 3.5 gm%	79 (73.8%)	120 (82.8%)	
Anatomical site of AD			
Umbilical region	57 (53.3%)	78 (53.8%)	0.9
Infra-umblical region	21 (19.6%)	29 (20%)	
Sub-xiphoid and epigastric regions	29 (27.1%)	38 (26.2%)	

AD, abdominal dehiscence; ASA, American Society of Anesthesiologists; COPD, chronic obstructive pulmonary disease; DM, diabetes mellitus; BMI, body mass index; HTN, hypertension

Supplementary Table 1 shows no statistical significance between the groups concerning departmental origin of the BA (0.4) and type of index operation (*p* = 0.1).

Operative details are presented in Table 2. Group A had a significantly longer median operative time than Group B (92 [IQR 88–100] vs. 89 [IQR 84–91] minutes, *p* < 0.001). There were no significant differences in estimated blood loss (*p* = 0.1) or need for blood transfusion (*p* = 0.09) between the groups. The etiology of AD differed significantly between the groups (*p* < 0.001), with “suture tearing” more common in group B (53.1% vs. 36.4%) and “infection” more common in group A (21.5% vs. 4.8%). The horizontal (0.07) and vertical (*p* = 0.06) dimensions of the initial fascial defects were comparable between groups.

**Table 2:** Operative details in both groups

	Group A (*n* = 107 )	Group B (*n* = 145 )	*p*-value
Possible aetiology of AD			
Tearing of the suture	39 (36.4%)	77 (53.1%)	< 0.001*
Infection	23 (21.5%)	7 (4.8%)	
Loose knot	5 (4.7%)	0 (0.00%)	
No explanation of aetiology	37 (34.6%)	50 (34.5%)	
Fascial necrosis	3 (2.8%)	11 (7.6%)	
Operative time (in minutes) (median, IQR)	92 (88-100)	89(84-91)	< 0.001*
Blood loss (in ml)			
< 500 ml	82 (76.6%)	122(84.1%)	0.1
> 500 ml	25 (23.4%)	23 (15.9%)	
Blood transfusion	25 (23.4%)	22 (15.2%)	0.09
Time between AD and surgical intervention (in days) (median, IQR)	3 (2–5)	3 (2–4)	0.009*
Horizontal length of AD (in cm) (mean, SD)	12.3 ± 2.6	12.2 ± 2.7	0.07
Vertical length of AD (in cm) (mean, SD)	15.2 ± 2.9	15.7 ± 2.3	0.06

AD, abdominal dehiscence. *Statistically significant

### Postoperative outcomes

Postoperative data and outcomes are summarized in Table 3. The mean hospital stay was significantly longer in Group A (11.2 ± 1.9 vs. 5.8 ± 1.5 days; *p* < 0.001). The overall complication profile, as assessed by the Clavien-Dindo classification, was different between the groups (*p* = 0.03), with a trend towards more severe complications (Grade IV) in Group B. Group A had a significantly higher incidence of seroma (22.4% vs. 11.0%, *p* = 0.01) and hematoma (3.7% vs. 0%, *p* = 0.01). SSI, ileus, and mortality rates were not significantly different between the groups. RAD was significantly lower in Group A (1.9% vs. 7.6%, *p* = 0.04) and occurred much later (19.5 ± 3.5 vs. 6.9 ± 1.7 days, *p* < 0.001) than in Group B. At the end of the 5-year follow-up period, the incidence of IH was significantly lower in Group A (6 patients (5.6%) versus 19 patients (13.1%) in Group B (*p* = 0.04)). IH in Group A developed significantly later (median 9 [IQR 6–26] vs. 6 [IQR 5–7] months, *p* = 0.03). They were also significantly smaller, with a median vertical defect length of 10 cm (7–12) versus 15.5 cm (12-17.7) in Group B (*p* = 0.02). IH in Group A is less likely to be complicated (0.9% vs. 10.3% of the entire cohort, *p* = 0.02).

**Table 3:** Postoperative data and outcomes in both groups

	Group A (*n* = 107 )	Group B (*n* = 145 )	*p*-value
Hospital stay (in days) (mean, SD)	11.2 ± 1.9	5.8 ± 1.5	< 0.001*
ICU		13(12.1%)	10(6.9%)
Clavien-Dindo classification (CD)			
CD-0	80 (74.8%)	103 (71%)	0.03*
CD-I	16 (15%)	27 (18.6%)	
CD-II	10 (9.3%)	5 (3.4%)	
CD-III	1 (0.9%)	2 (1.4%)	
CD-IV	0 (0.00%)	8 (5.5%)	
*Primary outcomes*			
SSI	8 (7.5%)	6 (4.1%)	0.2
Type of SSI			
Superficial	7 (6.5%)	5 (3.4%)	0.4
Deep	1 (0.9%)	1 (0.7%)	
Treatment of SSI			0.3
Drainage in bed	7 (6.5%)	5 (3.4%)	
Surgical or radiological drainage	1 (0.9%)	1 (0.7%)	
Seroma	24 (22.4%)	16 (11%)	0.01*
Hematoma	4 (3.7%)	0 (0.00%)	0.01*
Pneumonia (cough)	13 (12.1%)	6 (4.1%)	0.01*
Ileus	7 (6.5%)	6 (4.1%)	0.3
Myocardial infarction	2 (1.9%)	6 (4.1%)	0.3
Pulmonary embolism	1 (0.9%)	2 (1.4%)	0.7
Recurrent abdominal dehiscence (RAD)	2 (1.9%)	11 (7.6%)	0.04*
Time from surgery to RAD (days) (mean, SD)	6.9 ± 1.7	19.5 ± 3.5	< 0.001*
Time to RAD diagnosis (days) (mean, SD)	19.5 ± 3.5	6.9 ± 1.7	< 0.001*
Mortality	2 (1.9%)	5(3.4%)	0.4
*Secondary outcomes*			
Infected mesh	4 (3.7%)	0 (0.00%)	0.01*
Incisional hernia (IH)	6 (5.6%)	19 (13.1%)	0.04*
Time of IH development (in months) (median, IQR)	9 (6–26)	6 (5–7)	0.03*
Clinical presentation of IH			
Non-complicated IH	5 (4.7%)	4 (2.8%)	0.02*
Complicated IH	1 (0.9%)	15 (10.3%)	
Site of IH			0.1
Side of the wound	5 (4.7%)	13 (9%)	
Center of wound	1(0.9%)	6 (4.1%)	
Vertical length of defect of IH (in cm) (median, IQR)	10 (7–12)	15.5 (12-17.7)	0.02*
Horizontal length of defect IH (in cm) (mean, SD)	4 (4–5)	6 (4–8)	0.3

ICU, Intensive Care Unit; SSI, Surgical Site Infection; RAD, Recurrent abdominal dehiscence; IH, Incisional Hernia. *statistically significant

**Fig. 2:**
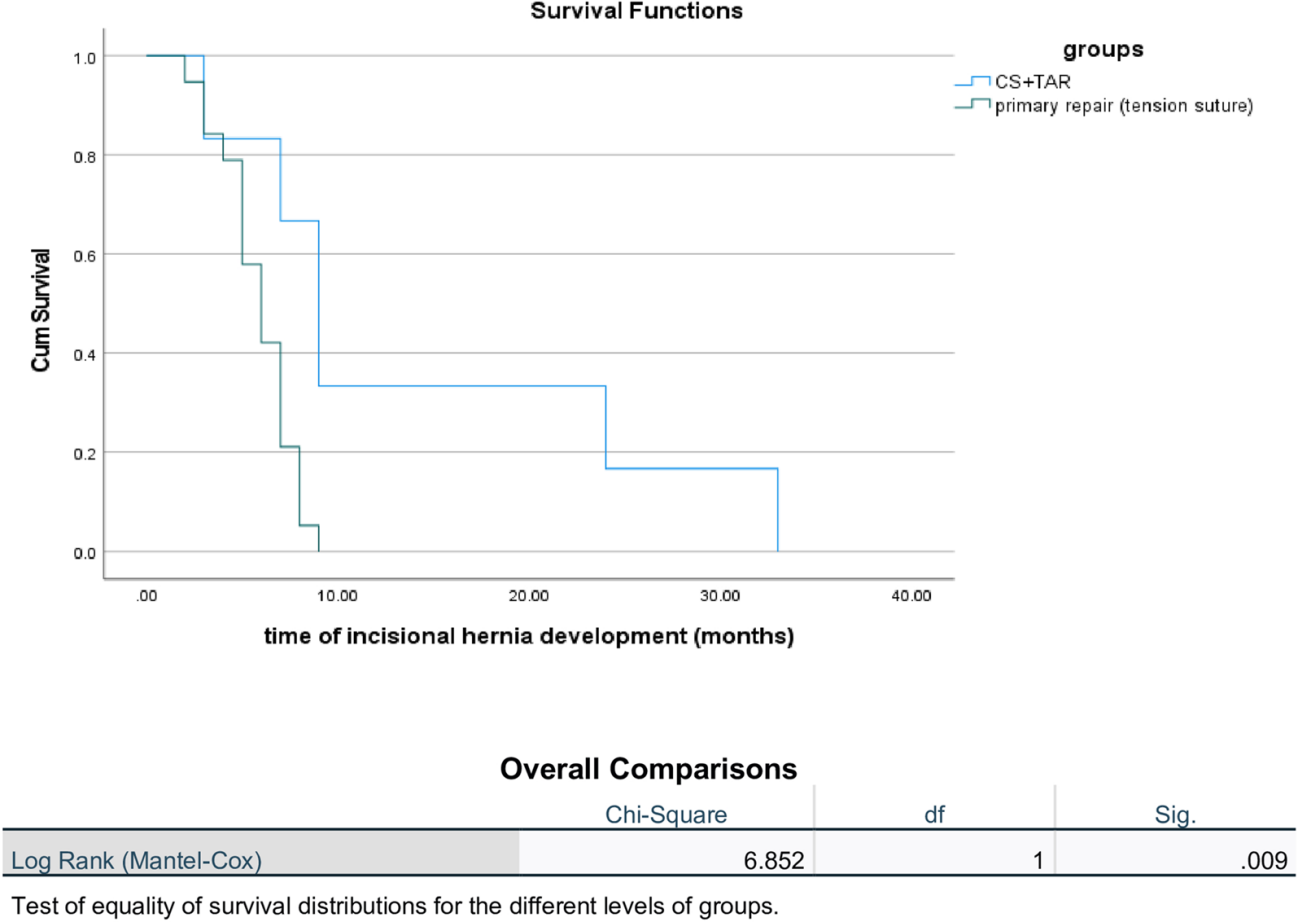
KM curve of incisional hernia free survival

The Kaplan–Meier curve in Fig. 2 provides the cumulative probability of remaining free from IH (IH-free survival).

1. *Group A* One year postoperatively, the curve for the CS + TAR+MA group plateaued at a high level, with a very shallow initial decline. After one year, the curve maintained its plateau for a sustained period before gradually declining. The log-rank test (Mantel-Cox) yielded a highly significant result (*P* = 0.009).
2. *Group B* The early postoperative year showed a steep initial decline in the curve. The curve continued to decline with a slightly reduced slope.

Univariate and multivariate logistic regression analyses were performed to identify independent predictors of IH development following surgical management of AD. The results are presented in Table 4. Multivariate analysis revealed that PS repair (OR 40.0, 95% CI 2.1–78.0; *p* = 0.01), SSI (OR 13.4, 95% CI 2.3–77.6; *p* = 0.004), pneumonia (OR 12.3, 95% CI 1.9–77.5; *p* = 0.007), high BMI (OR 2.9, 95% CI 1.06–4.1; *p* = 0.03), ileus (OR 16.6, 95% CI 2.2-121.9; *p* = 0.006), RAD (OR 10.7, 95% CI 1.5–73.3; *p* = 0.01), infected mesh (OR 14.6, 95% CI 1.8–117; *p* = 0.01), and old age (OR 1.07, 95% CI 1.006–1.15; *p* = 0.03) significantly increased the risk of IH after AD repair. Elevated serum albumin levels were protective (OR 0.1, 95% CI 0.0-0.7; *p* = 0.02).

**Table 4:** Univariate and multivariate logistic regression analysis to predict incisional hernia

	Univariate		Multivariate	
	OR (95% CI)	*P*-value	OR (95% CI)	*P*-value
Surgical technique (primary suture)	3 (1.1–8.1)	**0.003**	40 (2.1–78)	0.01*
Old age	1.08 (1.03–1.12)	**< 0.001**	1.07 (1.006–1.15)	0.03*
Sex	1.1 (0.5–2.8)	0.6	–	–
Vertical Length of AD	1.1 (1.001–1.2)	0.04*	1.1 (0.9–1.3)	0.2
DM	1.04 (0.3–2.9)	0.9	–	–
High Serum albumin	0.1 (0.05–0.3)	< 0.001*	0.1 (0.0-0.7)	0.02*
High BMI	1.8 (1.05–3.3)	0.03*	2.09 (1.06–4.1)	0.03*
Operative time	0.9 (0.9–1.02)	0.5	–	–
SSI	17.3 (5.3–55.7)	< 0.001*	13.4 (2.3–77.6)	0.004*
Seroma	0.7 (0.1–2.4)	0.5	–	–
Pneumonia(cough)	9.2 (3.2–26)	< 0.001*	12.3 (1.9–77.5)	0.007*
Ileus	20.8 (6.1–70.8)	< 0.001*	16.6 (2.2-121.9)	0.006*
RAD	4.6 (1.3–16.2)	0.01*	10.7 (1.5–73.3)	0.01*
Infected mesh	9.7 (1.3–72.7)	0.02*	14.6 (1.8–117)	0.01*

*Significant *P*-value; OR: Odds ratio; 95% CI: 95% confidence interval; AD : Abdominal dehiscence; DM: Diabetes Mellitus; BMI: Body Mass Index; SSI: Surgical Site Infection; RAD: Recurrent abdominal dehiscence

## Discussion

### Key findings

This study aimed to compare the early and late outcomes of PCS + TAR+MA (Group A) with those of PS (Group B). This study demonstrated that the surgical technique employed for AD management was a significant determinant of success. Group A had a higher incidence of seroma and hematoma (statistically significant) and a higher incidence of SSI (not reaching statistical significance). The Group A technique provided immediate superior mechanical stability, as demonstrated by a significantly lower RAD rate and delayed RAD onset compared to Group B. Our results show the superiority of the Group A technique, with a significantly lower incidence of IH and longer IH-free survival compared to Group B repair. Multivariate regression analysis identified that the Group B technique, postoperative complications (SSI, pneumonia, ileus), RAD, infected mesh, old age, and high BMI were risk factors for IH. A high serum albumin level is a protective factor.

### Primary (early) outcomes

While the Group A procedure was associated with a longer operative time and hospital stay, this is justified by the prevention of long-term sequelae. The prolonged hospital stay in Group A is likely multifactorial, attributable to the management of seromas (a known consequence of raising large flaps in component separation) [61]. It is crucial to note that Group B, despite shorter stays, suffered more severe complications (Clavien-Dindo IV). A seroma is a well-documented drawback of techniques that create large subcutaneous flaps for mesh placement and myofascial release. The dead space is a perfect environment for the accumulation of serous fluids. We found significantly higher rates of seroma (22.4%) and hematoma (3.7%) in Group A. Previous studies reported variable seroma rates of 11% [30] following PS and 4.7% [62] to 48% [12, 63–64] following CS techniques. The incidence of hematoma was significantly higher in Group A than in Group B due to the more extensive dissection, which increased the risk of bleeding and hematoma formation. We found a hematoma in Group A (3.7%), which was directly aligned with the findings of a previous study [30, 62]. The most critical point is regarding SSI. Despite the larger dissection and the presence of foreign material (mesh) in Group A, which was feared to lead to early complications, the SSI rate was not significantly higher. We believe that the use of mesh does not necessarily lead to higher SSI rates when combined with good surgical techniques and antibiotic prophylaxis, an opinion supported by previous studies [65, 66]. In contrast, other studies have shown that mesh use is contraindicated in acute settings [67].

Our results align with prior studies on abdominal wall reconstruction (AWR), indicating that increased procedural complexity is associated with early wound morbidity without a corresponding increase in SSI rates. Giordano et al. [61] found that performing a panniculectomy at the same time as abdominal wall reconstruction increased hospital stay length and the number of seromas but did not seem to increase SSI, RAD, or IH rates. We did not use panniculectomy in our study, and our results indicate that seroma and hematoma are not indicators of SSI but rather anticipated consequences of extensive myofascial dissection and the creation of dead space. Seroma following CS is manageable and is not a catastrophic complication. Additionally, early wound morbidity did not affect long-term outcomes (IH).

Postoperative pneumonia was significantly higher in Group A due to longer operative times and potentially more postoperative pain, which can impair pulmonary function, leading to atelectasis and pneumonia [68]. Ileus was not significantly different between the groups. Similar rates suggest that AD itself is a major driver of ileus rather than a specific repair technique. The overall distribution of complications (CD classification) was different, but the rate of major complications (CD III/IV) was lower in Group A (0.9% vs. 6.9%). The higher CD in Group B is notable and may be related to other severe sequelae. RAD is a serious complication occurring in 7.6% of group B and 1.9% of group A in our study, which is lower than the reported frequency of 4–13% [9] in PS, whereas RAD does not occur after component separation in another study [12]. The variation in RAD incidence is probably due to differences in patient inclusion, type of surgery (emergency or elective), type of mesh used, associated comorbidities, type of suture material, and PS repair technique (continuous or interrupted). Continuous monofilament sutures were used, as recommended by the EHS guidelines [10]. The time to RAD was much shorter in Group B (6.9 vs. 19.5 days, *p* < 0.001). The highest incidence of RAD was observed at the time of stitch removal and antibiotic cessation in both groups. Upon removal of the stitches, discontinuation of antibiotics may lead to relapse of the infection, and RAD becomes evident. In the postoperative period, the patients were in bed and began to move and defecate. All these factors increase intra-abdominal pressure. Additionally, PS attempts to approximate already compromised and often edematous tissues under high tension, which impairs microvascular flow, promotes ischemia, and creates a hostile wound environment conducive to failure, thereby impairing healing and predisposing patients to both early (RAD) and late failure (IH) [69].

In contrast, the Group A technique achieved tension-free closure reinforced with a mesh, reducing the risk of RAD. The significantly lower RAD rate in Group A (1.9% vs. 7.6%) indicates the immediate mechanical superiority of this technique. This aligns with a previous study that recommended using mesh reinforcement to prevent RAD [12] and another study that confirmed that tension-free repair was the cornerstone of success [70–71]. While the Group A technique can be applied with excellent success, the learning curve and the surgeon’s experience remain the greatest barriers to success. If CS is performed poorly, it can lead to RAD that are more challenging to repair than the initial defect itself. Our experienced surgeon was aware of the preoperative and perioperative critical steps required for operative success and avoided common pitfalls [59, 65].

### Secondary (delayed) outcomes

The group B technique is a less complex procedure, with fewer early complications (seromas and hematomas). However, it is biomechanically inferior. We found that IH was significantly higher in Group B due to the direct sequelae of failed healing under tension in PS. High tension on the suture line leads to tissue ischemia and failure, resulting in significantly higher long-term IH rates. Even if the closure initially holds, the strained tissue is prone to gradual elongation and herniation. In AD, the abdominal fascia is often of poor quality because of tearouts from the suture under tension [72]. Furthermore, patients with IH after AD repair often require a complex operation, with a risk of chronic pain, low quality of life, and a notable risk of recurrent IH [73–74]. These hernias are often larger and more prone to complications, leading to complex surgical procedures. In Group B, we strictly adhered to the guideline [10]. The Group A technique is a more complex procedure with a higher rate of early complications (seroma and hematoma). However, these complications were manageable. The Group A technique allows for large medial mobilization of the fascia edges on each side and is reinforced with a mesh. Therefore, Group A provides a more durable, tension-free, reinforced reconstruction and superior long-term durability, significantly reducing the risk of IH. Previous studies have concluded that mesh augmentation reduces the incidence of IH because the benefits of durable repair often outweigh the risks of mesh-related complications [75–76]. Our results (5.6% IH rate in Group A) are highly consistent and even superior to those of a previous study [34], strongly validating Group A’s superiority for durable repair, especially as experience with the technique increases.

The superior results of Group A over Group B regarding IH were validated using the Kaplan-Meier Curve (KMC). In Group A: KMC showed a few IH in the early postoperative period. The number of patients experiencing IH in the first year was significantly lower than that in Group B. After one year, the curve maintained its plateau for a sustained period before gradually declining. This suggests that IH in Group A was fewer in number and occurred later in the follow-up period. The log-rank test (Mantel-Cox), which compared the entire IH-free survival between the two groups, yielded a highly significant result (*p* = 0.009). This statistically confirmed that the differences in the timing and total number of IH between the two techniques were not due to chance. In Group B, the postoperative one-year survival curve showed a steep initial decline, indicating that a substantial number of IH occurred early in the follow-up period. The curve continued to decline, with a slightly reduced slope, indicating that IH continued to accumulate over time. However, the steep initial drop suggests that the number of new IH cases peaked immediately after surgery and then gradually decreased.

The Group A technique carries the risk of late-onset mesh infection. Infected mesh is a specific risk of the Group A technique, which can be a challenging complication to manage. However, the 3.7% rate was within the acceptable range reported in studies using meshes [12]. The use of mesh may be crucial for critically ill patients who experience severe setbacks from reoperations due to mesh complications. None of our patients required reoperation for mesh-related complications, whereas another study reported that 1/21 (5%) patients underwent partial mesh extirpation [12]. Therefore, we consider nonabsorbable mesh augmentation during CS + TAR to be safe and feasible, thereby reducing the need for reoperation after BA repair.

### Risk factors for IH after AD repair

The multivariate results showed that the Group B technique was associated with 40 times higher odds of IH, similar to a previous study [6]. Older age is a significant independent predictor of decreased tissue quality and healing capacity. Atherosclerotic changes in blood vessel walls result in reduced tissue perfusion and increased susceptibility to infection, often associated with decreased immunity. The older age group likely complained of chronic cough, constipation, dysuria, anemia, hypoproteinemia, and multiple vitamin deficiencies. Postoperative complications such as cough, vomiting, and respiratory system infection are more common in the older age group. Hermosa et al. confirmed the correlation between old age and IH [77]. The albumin level is a measure of a patient’s nutritional status. In our logistic regression analysis, higher albumin levels were protective. Malnutrition contributes to dehiscence by impairing collagen and ground-substance synthesis at the wound site, thereby delaying healing, reducing wound strength, and increasing AD. Our results are similar to those of a previous study [78].

SSI increased the odds of IH by 13 times. Wound infection disrupts healing, destroys tissues, causes sloughing of stitches, and separates the rectus sheath [79]. Pneumonia causes coughing, which dramatically increases intra-abdominal pressure and stress during repair, leading to 12 times higher odds of IH. Pneumonia usually occurs due to post-anesthetic respiratory tract infection, leading to increased intra-abdominal pressure. Thus, tension over the sutured wound can cause IH [68]. Similarly, postoperative abdominal distention and vomiting due to postoperative ileus increase the tension on the wound, leading to IH [80]. In addition, increased intra-abdominal tension due to ileus plays a significant role in the incidence of IH, which is why, upon releasing this tension in Group A, a significant decrease in the incidence of IH occurred. Higher BMI causes more strain on the abdominal wall and is associated with worse wound healing. It is a modifiable risk factor for patients. Previous studies have reported obesity as a risk factor for IH [81–83]. RAD severely compromises the abdominal wall, which makes it more likely to develop into IH later. While the mesh prevents hernias, if it becomes infected, it can no longer provide support, thereby increasing the risk of failure.

### Limitations

The retrospective study design and the non-random choice of technique thus introduce a potential selection bias. However, baseline comparability of the groups mitigated this concern to some extent. To ensure reliable data, we included only patients who began treatment in our departments with the same wound care specialists, enabling us to collect complete and consistent records. A prospective randomized controlled trial is the next step in confirming these results. Calculation of the Charlson Comorbidity Index (CCI) could provide an additional layer of global risk stratification. Given the retrospective nature of the study and incomplete availability of all weighted CCI components (e.g., malignancy stage, cerebrovascular disease severity), a formal CCI calculation could not be performed reliably without risking misclassification. Subspecialized emergency surgical teams performed the procedures and adhered to a well-defined strategy for high-risk emergency surgical patients, including standardized preoperative, intraoperative, and postoperative protocols, as well as documented standards for the surgical treatment of AD, thereby reducing inter-center variability.

## Conclusion and recommendations

The group A technique for AD repair was associated with a significantly reduced risk of IH and RAD compared with the PS group. Despite a longer operative time and hospital stay, Group A provides a more durable and definitive reconstruction, making it a preferred surgical strategy for AD. Group A transformed a catastrophic event into an opportunity for definitive repair, significantly reducing the need for future, more complex surgeries related to recurrence and IH. However, a higher seroma rate is a complication but a manageable cost for long-term benefits. Postoperative risk factors for IH can be decreased by avoiding increased intra-abdominal pressure, such as coughing or vomiting, and by measures to avoid excessive straining during urination or defecation. Conditions such as ileus or urinary bladder distention should be promptly managed using nasogastric decompression or catheterization. Proper preoperative skin preparation followed by aseptic and antiseptic application can effectively reduce the microbial burden and lower the risk of SSI. Additionally, optimizing patients’ nutritional status improves postoperative outcomes.

## Supplementary Information

Below is the link to the electronic supplementary material.

Supplementary Material 1

## Data Availability

Data are available from the corresponding author upon reasonable request.

## Declarations

Patients provided informed consent to publish their data and photographs.
