# Supplementary material for: Early and late outcomes of component separation with transversus abdominis release with mesh augmentation versus primary suturing for the management of abdominal dehiscence: a retrospective comparative study
Source: World J Emerg Surg. 2026 Mar 31;21:22. doi: 10.1186/s13017-026-00690-2 (PMC13064292; doi:10.1186/s13017-026-00690-2)
Supplement: Supplementary file 1 — Supplementary Material 1 [file 13017_2026_690_MOESM1_ESM.doc]

Supplementary table 1: departmental origin of the abdominal wall dehiscence and type of index operation
		Group A
(n=107 )	Group B
(n=145 )	p-value	
Departmental origin of abdominal dehiscence (AD) 		General, trauma, and GIT surgery	79 (73.8%) 	113 (77.9%) 	0.4	
		Obstetrician surgery 	20 (18.7%) 	26 (17.9%) 		
		Vascular surgery 	8 (7.5%) 	6 (4.1%) 		
Previous index operation		General, trauma, and GIT surgery	0.1	
	Appendix surgery	Emergent lower Midline for perforated appendicitis	12(11.2%)	19(13.3%)		
		emergent lower Midline for converted lap perforated appendicitis	3(2.7%)	2(1.4%)		
		emergent Midline for perforated appendicitis	0(0.00%)	2(1.4%)		
	Colorectal disease and cancer	elective lower Midline for the anterior resection of the cancer rectum	1(0.9%)	0(0.00%)		
		emergent Midline laparotomy for obstructed cancer colon	1(0.9%)	0(0.00%)		
		elective lower Midline for sigmoidectomy for diverticulosis coli	1(0.9%)	0(0.00%)		
		elective Lower Midline for colovesical fistulectomy	1(0.9%)	0(0.00%)		
		elective Lower midline incision for rectovaginal fistula	1(0.9%)	0(0.00%)		
		elective Midline for cancer caecum	9(8.4%)	14(9.7%)		
		elective Midline for cancer of the right colon	1(0.9%)	0(0.00%)		
		Emergent Midline for cancer colon	0(0.00%)	1(0.7%)		
		Emergent lower Midline for perforated diverticulitis	1(0.9%)	0(0.00%)		
		elective Lower Midline for rectovaginal fistula	1(0.9%)	4(2.8%)		
		Emergent Midline for volvulous sigmoid resection	1(0.9%)	0(0.00%)		
		elective Midline for cancer transverse colon	1(0.9%)	3(2.1%)		
		Emergent Midline for obstructed cancer caecum	0(0.00%)	1(0.7%)		
		elective Midline for converted laparoscopic cancer caecum	0(0.00%)	2(1.4%)		
	Gallbladder surgery	Emergent upper Midline for converted laparoscopic  cholecystectomy	2(1.8%)	0(0.00%)		
		elective Upper Midline for converted laparoscopic cholecystectomy and splenectomy for blood disease	1(0.9%)	0(0.00%)		
		Emergent upper Midline for perforated gallbladder	1(0.9%)	3(2.1%)		
		Emergent Midline for biliary peritonitis following lap cholecystectomy	1(0.9%)	0(0.00%)		
	Abdominal cyst	elective Midline for abdominal mesenteric cyst	2(1.8%)	0(0.00%)		
	Pancreatic surgery	Emergent Midline exploration for pancreatic necrosis debridement	3(2.8%)	1(0.7%)		
		elective Upper Midline for treatment of pancreatic pseudocyst	2(1.8%)	0(0.00%)		
	Gastric surgery	Emergent Midline for converted laparoscopic perforated peptic ulcer	2(1.8%)	0(0.00%)		
		Emergent Midline for perforated peptic ulcer	8(7.5%)	13(8.9%)		
		elective Midline for excision of gastric leiomyoma	1(0.9%)	0(0.00%)		
		elective Upper Midline for converted lap  sleeve gastrectomy	1(0.9%)	0(0.00%)		
		elective  Midline incision for gastric leomyoma	0(0.00%)	1(0.7%)		
	Small intestine surgery	emergent Midline for Small intestinal resection anastomosis for intestinal obstruction and strangulation	2(1.8%)	0(0.00%)		
		Emergent Midline for perforated typhoid ileal ulcer perforation	2(1.8%)	5(3.5%)		
		Emergent Midline for mesenteric vascular occlusion	6(5.6%)	12(8.3%)		
	Trauma surgery	Emergent Midline exploration for gunshot	1(0.9%)	0(0.00%)		
		emergent Midline laparotomy for internal hemorrhage	10(9.3%)	27(18.7%)		
	Pelvic surgery	Emergent lower Midline for pelvic abscess	0(0.00%)	3(2.1%)		
		Vascular surgery(15)		
		Emergent Midline for open abdominal aortic aneurysm surgery	1(0.9%)	0(0.00%)		
		Elective open Midline for abdominal aortic aneurysm surgery
	7(6.5%)	6(4.2%)		
		Obstetrician surgery		
		Emergent lower Midline for intra-abdominal abscess following cesarean section	7(6.5%)	6(4.1%)		
		Emergent lower Midline for converted laparoscopic hysterectomy due to bleeding during myomectomy	1(0.9%)	1(0.7%)		
		Emergent lower Midline for hysterectomy due to bleeding following CS	2(1.8%)	5(3.4%)		
		elective Lower Midline for TAH+BSO for ovarian cancer	8(7.4%)	10(6.8%)		
		elective Midline incision for ovarian cystectomy	1(0.9%)	1(0.7%)		
		Emergent lower Midline for tubo-ovarian abscess	1(0.9%)	3(2.1%)		
